# 

**Published:** 1825-04-01

**Authors:** 


### BIBLIOGRAPHICAL RECORD;

OR,

*Works received for Review between the 20th of December, 1824, and the 15th of March, 1825.*

1. An Estimate of the true Value of Vaccination as a Security against Small Pox. By T. M. GREENHOW, Member of the Royal College of Surgeons; Surgeon to the Lying-in Hospital, &c. Octavo, pp. 75. December, 1824.

☞ *This little work is very well calculated for the general reader, to whom it is principally addressed.*

---

2. Revue Medicale, Française et Etrangère; et Journal de Clinique de l'Hotel Dieu et de la Charité. Novembre, 1824. *(In exchange.)*

---

3. A Treatise on Moxa, as applicable more particularly to Stiff-joints: illustrated by Cases and Plates: with some general Observations on Spinal Diseases. By JAMES BOYLE, Esq. Surgeon to the Middlesex Infirmary—Author of a Treatise upon the Epidemic Cholera of India, &c. &c. Octavo, pp. 168. Callow and Wilson, 1825.

---

4. An Analysis of Medical Evidence: comprising Directions for Practitioners, in the View of becoming Witnesses in Courts of Justice; and an Appendix of professional Testimony. By JOHN GORDON SMITH, M.D. Octavo, pp. 386. Underwoods, January, 1825.

---

5. Revue Medicale, Française et Etrangère; et Journal de Clinique de l'Hotel Dieu et de la Charité. Decembre, 1824.

---

6. The Westminster Review. No. V. January, 1825.

☞ *Among the numerous articles in this rising work, we notice a laboured, and certainly an able one, on the subject of Quarantine, Contagion, &c. While we differ from the writers of that article in the view which they take of contagious diseases generally, we award to them the full meed of praise for the talent which they evince in the construction and support of their arguments.*

---

7. Dissertatio Chemico-Physiologica de Sanguine, ejusque mutationibus. Auctore, CAROLO J. B. WILLIAMS, M.D. Ed. 1824.

☞ *This is an ingenious Thesis, and contains several curious experiments illustrative of the subject discussed.*

---

8. An Essay on Venereal Diseases, and the Uses and Abuses of Mercury in their Treatment. Illustrated by Drawings of the different Forms of Venereal Eruptions. By RICHARD CARMICHAEL, M.R.I.A. Vice President of the Royal College of Surgeons in Ireland, and one of the Surgeons of the Richmond Surgical Hospital, &c. &c. Octavo, pp. 376, with five beautifully coloured Plates. (Second Edition.) London and Dublin, March, 1825.

☞ *This valuable Treatise (which is, in fact, an almost entirely new publication) will be certainly analyzed in our next number.*

---

9. A Compendious System of Midwifery, chiefly designed to facilitate the Inquiries of those who may be pursuing this Branch of Study. Illustrated by occasional Cases. With Engravings. By W. P. Dewees, M.D. Lecturer on Midwifery, &c. Octavo, pp. 602. Plates. Philadelphia, 1824.

☞ *This is a very valuable compilation, containing a good deal of original matter, and evincing very considerable research and judicious observation.*

---

10. Revue Medicale Française et Etrangère, et Journal de Clinique de l'Hotel Dieu, &c. Jan. 1825.

*In exchange.*

---

11. Report of the Physician of the Small-Pox and Vaccination Hospital. By George Gregory, M.D. Octavo, sewed, pp. 15. February, 1825.

☞ *See Extra Limites.*

---

12. System of Anatomical Plates, with descriptive Letter-Press. By John Lizars, F.R.S. &c. Lecturer on Anatomy and Physiology, Edin. Part VII. The Brain. First Portion, coloured after nature. 1*l.* 1*s.* coloured.

☞ *This fasciculus contains several plates of the brain and spinal marrow, most beautifully coloured, and, in fact, exceeding, in fidelity and spirit, all the parts that have gone before.*

*In the Bulletin des Sciences Medicales, for January of the present year, we observe the following notice of this meritorious performance;*

" *Cet ouvrage se continue avec activité. Les planches sont coloriées. Les quartre parties que nous avons sous les yeux nous paraissent propres à rendre familière aux jeunes gens qui se destinent à l'art de guérir, l'étude et la connaissance approfondie des systêmes osseux, musculaire, nerveux, et vasculaire, du corps humain.*"

*Such a testimony from foreigners must be gratifying to the author and convincing to the public. Mr. Lizars apologises for the delay in the appearance of the present fasciculus—a delay occasioned by the great care necessarily taken up with the colouring of the plates of this very minute and important organ—and which the author found to be indispensably requisite, in order to give a clear and distinct development of its various portions. These considerations are sufficient reasons why this and the next fasciculus should appear entirely coloured.*

*We wish Mr. Lizars a continuation of that success which he eminently deserves.*

---

13. Observations on the Use of the Colchicum Autumnale in the Treatment of Gout; and on the proper Means of preventing the recurrence of that disorder. By Charles Scudamore, M.D. F.R.S. Member of the Royal College of Physicians, &c. &c. &c. Octavo, pp. 116. 1825.

☞ *In our next.*

---

14. Elements of Medical Jurisprudence. By Theodore Romeyn Beck, M.D. &c. &c. &c. Second Edition, with Notes, and an Appendix of original Cases and the latest Discoveries. By William Dunlop, Esq. &c. Lecturer on Medical Jurisprudence. Octavo, pp. 640. London, March, 1825.

☞ *We gave our meed of praise to the original American Edition. The present republication in this Country is much improved, and, as it stands, forms one of the best systems of medical jurisprudence which we possess. We shall take some further notice of this edition in our next.*

---

15. An Account of the Disease lately prevalent at the General Penitentiary. By P. MERE LATHAM, M.D. Fellow of the Royal College of Physicians, and Physician to St. Bartholomew's Hospital. Octavo, pp. 286. London, March 8th, 1825. ☞ *In our next.*

---

16. An Elementary Description of the Anatomy and Physiology of the Brain, Viscera of the Thorax, Abdomen, &c. with corresponding Questions, designed more particularly for the Use of Gentlemen preparing for Examination at Apothecaries' Hall, also for junior Students. By a Licentiate of the Society of Apothecaries. London, March 1825. Price, 2s.

---

17. A Letter to the Right Honorable the Earl of Egremont, on the subject of a Sussex County Hospital at Brighton. By W. KING, M.D. Fellow of the Royal College of Physicians, &c. Brighton, March 1825. Pp. 16, price 6d.

☞ *This letter, which is well written, contains many suggestions which we hope will be taken into consideration by the managing committee of the new Institution at Brighton.*

---

18. Part Second of a Series of Elementary Lectures on the Veterinary Art; wherein the Anatomy, Physiology, and Pathology of the Horse are essayed on the General Principles of Medical Science. By WILLIAM PERCIVALL, Member of the Royal College of Surgeons in London, &c. &c. Octavo, pp. 558. Longmans, 1825.

☞ *See what we have said of Part the First.*

---

19. Proceedings at the Sixth Anniversary Meeting of the Hunterian Society, held on 9th February, 1825.

☞ *We are glad to observe that this Society flourishes.*

---

*** NOTE.—Authors and publishers will readily appreciate the importance of having their works recorded, with full length titles, on this list, which stands as a perpetual advertisement so long as the Journal lasts, and as far as it extends. The republication of the Journal in America enhances the advantages of the BIBLIOGRAPHICAL RECORD, on which no work can be entered, unless transmitted, free of expence, to the Editor, under sealed cover to the publishers, or in any other way most convenient to the parties concerned.

---

MEDICO-CHIRURGICAL REVIEW.

*(American Edition.)*

All the back Numbers of this Journal, from No. 1 to No. 16, inclusive, have now been imported (by permission of the Editor) by Messrs. Burgess and Hill, who can supply gentlemen with complete sets, according to order.

☞ *The American edition is printed verbatim from the London edition, and detached numbers will correspond so as to complete imperfect sets without any deformity to the work.*

55, *Great Windmill Street, March 20th*, 1825.

### *Additional Subscribers since last Quarter.*

A.

Allen, Dr. Epping Forest Asylum, Leopard's-Hill Lodge, Loughton, Essex.

Andrews, Mr. W. S. Surgeon, Richmond.

Anstice, — Esq. St. John's Wood Road.

B.

Blest, Dr. William C. Member of the Medical Society of Trinity College, Dublin, of the Medico-Chirurgical Society of Edinburgh, &c. Santiago, Chili.

Burnett, Dr. William, Member of the Royal College of Physicians of London, Medical Commissioner of His Majesty's Navy, &c. Welbeck Street, Cavendish Square.

C.

Cave, Thomas, Esq. Surgeon, Salisbury.

Coates, Amos, Esq. Surgeon, York.

Corban, Francis, M.D. Member of the Royal College of Surgeons of London, Glandore Dispensary, County of Cork.

D.

De La Hooke, Wm. Esq. Plympton, Devon.

Dimond, Mr. W. B. Weston House, Weston Street, St. Pancras.

E.

Elsegood, Mr. Upper Brook Street.

G.

Granville, Dr. Augustus, B. &c. Grafton-street, Berkeley Square.

Gullan, Mr. Hon. East India Company's Service, George Street, Euston Square.

H.

Hardman, Dr.

I.

Ingram, Mr. James, Buenos Ayres.

L.

Lomas, Mr. Thomas, Surgeon, Belper, Derbyshire.

Lucas, Mr. Surgeon, &c. Charles Street, Soho.

M.

M'Morris, Joseph, Esq. Superintendant of Vaccination, Guzerat District, Bombay Establishment.

M'Morris, John, Esq. Assistant-Surgeon, H.M. 4th Regiment of Cavalry, at Kaira, Bombay Establishment.

Martin, Mr. Robert, Surgeon, Holbrook, near Ipswich.

Morrah, Mr. Surg. Sloane Street.

Muriel, Mr. Borough.

N.

Nursaw, Mr. Thomas, Student.

P.

Pavy, Mr. Woodstock.

R.

Rea, Dr.

Rodolph, Francis, Esq. Rio Janiero.

S.

Shaw, Mr. Surgeon, Aston, Cumberland.

Sluder, Mr. Charles, Student.

Spry, J. H. Esq. Charter House Square.

W.

Warren, Mr. Charles, Surgeon, to Chili.

Warwick, Mr. J. B. Southwell, Notts.

Watson, Dr. Bernard Street, Russell Square.

Watson, Mr. John, Surgeon, Beomopfield, Durham.

Wilson, Mr. Robert, to Callao, South America.

Woolaston, Mr. Surg. Clapham.

---

☞ Mr. Brande's Manual of Pharmacy was received too late for insertion in the Bibliographical Record.

### INDEX.

Abortion, Dr. Musgrave's case of 513
Abortion, culpable instance of.. 528
Abscess in the stomach........ 180
Abuse of purgatives in piles.... 289
Acetate of morphine in Rheumatism ...................... 531
Acid (lactic) in the human stomach ...................... 145
Acne, Mr. Plumbe on.......... 46
Air, fatal introduction of, by a vein ...................... 218
Alimentary canal, perforations of 170
Alison, Dr. on scrofula.......... 98
Amesbury, Mr. on non-union of fractures .................. 249
Amputation of the lower jaw.... 210
Amputation (successful) at the hip-joint .................. 225
Analecta, or little periscope .... 248
Analecta Minora .............. 530
Anatomy of the Skin .......... 30
Angina pectoris, Dr. Hosack on.. 59
Angular projection of the spine.. 141
Animal putrefaction, fatal instance of.......................... 202
Anomalous gout, M. Bayle...... 187
Antimonium tartarizatum, remarks on .................. 357
Apoplexy, tendency of puerperal women to .................. 517
Arnott, Mr. on ptyalism........ 153
Ascension, fatal fever at........ 5
Ascites, treated by compression.. 530
Ascites and pregnancy combined 506
Asthma, fatal from affection of glottis....................... 458
Auscultation, Laennec and Forbes on ........................ 61
Avenbrugger on Percussion .... 65
Balfour, Dr. on Hernia ........ 212
Balfour, Dr. his striking cures.. 234
Bampfield, Mr. on the spine.... 112
Bann, fever in................ 1
Basis Cranii, imagined fracture of 466
Bedford man of war, fever in .... 10
Belcher, Dr. William, on enlarged tonsils...................... 317
Biliary calculus, Dr. Craigie on.. 159
Bladder opened by Mr. A. C. Hutchison........................ 224
Blundell, Dr. his physiological researches.................... 400
Borate of potassa, Dr. Hamilton's catholicon.................. 515
Bourdon, M. on organic disease of stomach.................. 174
Boyle, Mr. on Moxa............ 377
Bronchocele, iodine in........ 229
Brook, Dr. on liver-cough...... 208
Buchu leaves, in diseases of the bladder .................... 485
Burnett, Dr. on fever.......... 1
Byron, Lord—dissection of...... 164
Cæsarian section, substitute for.. 522
Calculus, biliary, a large one passed 159
Callow, surgeon, on abscess of the stomach.................. 180
Calvert, Mr. on hæmorrhoids .. 273
Cancer, correction of its fetor.... 249
Carmichael, Mr. on tetanus .... 193
Carswell, Mr. on melanosis.... 154
Carter, Dr. on occult disease of the chest ................ 161
Case of supposed disease of the heart ...................... 461
Castor oil, substitute for........ 249
Cayol, M. his views of fever .... 463
Cellular texture, inflammation of 335
Cerebral affection.............. 192
Chronic diseases, percussion in.. 70
Chronic enlargement of the tonsils ...................... 217
Circulating function, Dr. Uwins's ideas of .................... 385
Classification of cutaneous diseases ...................... 45
Cleverly, Dr. his death ........ 256
Colchicum in rheumatism...... 430
Colon, cases of accumulations in the .......................... 489
Compendium of medical practice 382
Compression, its effect in ascites 500
Congestive fever in the sea ...... 240
Contagion, definition of........ 389
Contagious glanders, cure of.... 249
Contraction of the anus........ 283
Conversion of disease, Dr. Crampton....................... 197
Convulsions peculiar to children 493
Cooke, the tragedian, dissection of.......................... 61
Corvisart, on percussion........ 65
Crampton, Dr. on conversion of disease .................... 197
Crampton, Dr. on opium poisoning ........................ 235
Crown Assurance Company, prospectus of .................. 556
Cubebs, volatile oil of.......... 532
Cullen, Mr. on melanosis ...... 154
Curvatures of the spine, essay on 112
Cutaneous diseases, works on .. 29
Cuticle, anatomy and functions of 30
Cynache trachealis ............ 181
Dangers of iodine.............. 233
Datura stramonium in nervous complaints.................. 491
Dawson's, Dr. practice of physic 382
Death, sudden, in an operation.. 218
Delpech's amputation at the hip-joint ...................... 225
Diabetes, Dr. Sharkey on ...... 237
Diffuse inflammation, Dr. Duncan on ........................ 335

Discharge, hæmorrhoidal, nature of........................ 274
Distortions of the spine, Mr. Bampfield on ............... 112
Dropsy in pregnancy, curious case of ...................... 523
Drug, Dr. on the etiology of goitre 442
Duncan, Dr. on phlebitis...... 161
Duncan, Dr. on diffuse inflammation...... ............... 335
Dupuytren, operations by...... 219
Dysentery, Dr. O'Halloran's treatment of.................... 234
Dysentery, employment of tobacco in ........................ 495
Dyspnœa, fatal cases of ........ 444
Emetics, their efficacy in hæmatemesis ....................... 481
Emplasto - dermic treatment of diseases ..................... 248
Epilepsy, on the pathology of .. 205
Epilepsy, tartar emetic ointment in 486
Etiology of cutaneous diseases.. 42
Etiology of scrofula............ 98
Etiology of hæmorrhoids........ 285
Etiology of goitre............... 442
Excurvation of the spine........ 114
Expectoration in phthisis, acid & alkaline ..................... 452
Extinction of fire, recipe for.... 530
Extirpation of the ovaria...... 215
Extract of lettuce, a substitute for opium .................. 531
Extra-limites .................. 536
Extravasations in the chest...... 71
Eye, its indications in disease.... 425
Fever, eclectic article on........ 1
Fever, Dr. Hosack on.......... 54
Fever, caused by panic........ 148
Fever, intermittent, partial .... 201
Fever from animal putrefaction 202
Fever, Laennec's report on....... 240
Fever, congestive, in the sea.... 249
Fever, remarks on, by Dr. Dawson ........................ 394
Fissures, hæmorrhoidal ........ 281
Foderée, M. his case of abortion.. 529
Forbes, Dr. John, on the stethoscope, &c. .................... 61
Forceps for extracting teeth perpendicularly .................. 560
Fractures, on non-union of...... 249
Gairdner, Dr. on perforations of stomach ..................... 170
Gairdner, Dr. on purpura hæmorrhagica ...................... 193
Gangrene, at the mouth of the uterus in puerperal fever .... 511
Gastritis, a peculiar species of .. 173
Gastritis, Dr. Uwins's view of .. 391
Gastrodynia, its history and treatment ...................... 396
Gastro-enteritis, supposed case of 463
General practitioner, observations on the...................... 246
Glanders, on the cure of........ 249
Glossitis, Dr. Hosack on........ 55
Glottis, fatal affection of........ 458
Goitre, efficacy of iodine in...... 229
Goitre, its etiology ............ 442
Gout, Dr. Hosack on........... 58
Gout, anomalous, M. Bayle...... 187
Grave, fatal effects from opening a 202
Graves, Dr. on free acid in the stomach ................... 145
Greenhow, Mr. on medical remuneration................... 246
Gregory, Dr. his small pox report 551
Hæmatemesis cured by emetics.. 481
Hæmorrhage into the bladder .. 224
Hæmorrhoids, Mr. Calvert and others on ................. 273
Hamilton, Dr. on panic fever.. 148
Hamilton, Mr. on animal effluvia 202
Head, case of severe affection of the ........................ 490
Henning, Dr. his dissection .... 457
Hepatic phthisis .............. 184
Hepatization of the lungs—what 72
Herniæ, curious treatment of.... 211
Hernia, Mr. Yeatman's treatment of ........................ 467
Hewson, Mr. on ophthalmia .... 305
Hip-joint amputation, successful 225
Hosack, Dr. miscellaneous papers 53
Hospital reports of M. Laennec.. 240
Hospital reports, by M. Cayol .. 460
Hospital reports by Dr. Hosack.. 482
Hutchison, Mr. A. Copland, operation by.................. 224
Hydrocephalus cured by mercury 501
Hydrophobia, supposed fatal case of ........................ 497
Hydrophobia prevented by mercury ..................... 499
Idiotcy from nervous injury .... 469
Idiosyncrasy of individuals, M. Martinet on ................ 433
Incurvations of the spine ...... 132
Infants, ophthalmia of, Mr. Ryall 220
Inflammation of veins—Dr. Duncan ........................ 161
Inflammation of nerves—M. Martinet ...................... 165
Inflammation of appendix cœci.. 200
Inflammation, hæmorrhoidal.... 280
Inflammation, Dr. Uwins's remarks on ................... 387
Inflammation of veins, Dr. Chapman's case of............... 447
Inflammation of veins, Mr. Hodgson's case of .............. 449
Inflammation and irritation of the Bronchiæ ................. 451
Intermittent, partial .......... 201
Intermittents denied by Dr. Mills 16
Intestines, perforation of ...... 182
Insanity, Dr. Willis on ........ 360
Intus-susceptio, a remarkable case of ........................ 460

Iodine, memoirs on the use of .. 229
Iodine, dangers of ............ 233
Iritis, Dr. Robertson on ........ 468
Iritis, Mr. Melin on............ 471
Iron, remarks on by Mr. Phillips 358
Irritable uterus................ 512
Italian remedies .............. 530
Jaws, anchylosis of ............ 472
Kellie, Dr. on a peculiar spasm in children ............... 454
Kent man of war, fever in...... 10
Kinglake, Dr. on hernia........ 211
Knife cut out from the stomach.. 477
Knife-swallowing, a case of...... 476
Kolley, Dr. on iodine .......... 229
Laceration of the womb, case of 406
Lactic acid (free) in the stomach 145
Laennec on auscultation........ 61
Laennec's report on fever .. ... 240
Laryngitis, Mr. Carmichael on .. 223
Lateral curvature, Mr. Bampfield on ........................ 123
Ligature and knife, comparison of for piles.................. 292
Lightning, effects of .......... 149
Lightning, effects of .......... 199
Lithotomy, recto-vesical........ 474
Liver-cough, Dr. Brooke on .... 208
Lizars, Mr. on extirpation of the ovaria..................... 215
Louis, M. on a peculiar gastritis 173
Macauley, Dr. on the effects of lightning .................. 199
Maclean, Dr. on quarantine..... 1
Maclelland, Dr. amputation of the lower jaw ............. 210
Martinet, M. on individual idiosyncrasy ...................... 433
Martinet, Dr. on neuritis ...... 165
Medical remuneration.......... 246
Melanosis, Messrs. Cullen and Carswell on ............... 154
Mental derangement, Dr. Willis on ........................ 360
Metastasis of rheumatism to the brain ...................... 431
Miguel, Dr. on the dangers of iodine...................... 233
Mills, Dr. on fever and inflammation .................... 14
Moriarty, Sir T. on hepatic phthisis................... 184
Montegre, M. on hæmorrhoids .. 273
Mortal dyspnœa, cases of ...... 444
Moxa, Mr. Boyle on............ 377
Musgrave, Dr. his case of abortion 513
Myers, Dr. on cynanche trachealis ................... 181
Nerves of the spine, anatomy of.. 151
Nerves of the spine, inflammation of, Martinet ............... 165
Nervous pain, hæmorrhoidal.... 282
Nervous injury, a cause of idiotcy 469
Nervous pains, datura stramonium in ........................ 492
Nitrate of potash in dropsies.... 531
Nitre extracted from the stomach 487
North, Mr. on peculiar spasms in children.................. 452
Occult disease of the chest—Dr. Carter .................. 161
O'Brien, Dr. on sulphate of quinine ..................... 227
O'Halloran, Dr. on dysentery.... 234
O'Halloran, Dr. on ophthalmia.. 305
Operation, sudden death in .... 218
Ophthalmia, Mr. Ryall on....... 220
Ophthalmia, Dr. O'Halloran on.. 305
Ophthalmia, Mr. Melin on...... 470
Opium, in puerperal irritability.. 504
Opium, poisoning by .......... 235
Œdemato-phlegmonous neuritis 525
Ovaria, extirpation of .......... 215
Pains, hæmorrhoidal .......... 281
Panic, the cause of fever........ 148
Paracentesis thoracis .......... 96
Paracentesis thoracis .......... 213
Paracentesis capitis, Mr. Money's case of ................... 478
Paracentesis Thoracis, Morand's case of .................. 480
Pathology of scrofula, Dr. Alison on ...................... 99
Pathology of epilepsy, Dr. Reid.. 205
Payne, Dr. on puerperal fever .. 243
Peritonitis, Laennec's report on.. 242
Peritonitis, M. Tacheron on .... 332
Pericarditis, M. Tacheron on.... 326
Pericardium, tympanitic state of 460
Peripneumony, percussion in.... 69
Perforation of the intestines.... 182
Percussion, Avenbrugger, &c. on 65
Perforations of the stomach, Dr. Gairdner on ............... 170
Persulphate of quinine, formulary of ...................... 483
Pharmacopœia, Mr. Phillips on.. 256
Phrenology, Mr. Coombe on.... 251
Phlegmatia dolens, Dr. Hosack on 57
Phillips, Mr. his pharmacopœia.. 356
Phthisis, hepatic .............. 184
Physic, Drs. Uwins and Dawson on ...................... 382
Phosphate of soda in diabetes.... 237
Pinch of snuff, pathology of .... 250
Plague, symptoms of, from animal effluvia .................. 202
Pleurisy, Laennec's report on.... 241
Poisoning by opium............ 235
Poison preserves a human stomach ................... 250
Pneumonia, on the treatment of 236
Preservation of dead subjects.... 248
Pregnancy and ascites, cases of.. 506
Preternatural presentation, case of 507
Pressure on the perineum in labour ..................... 530
Prolapsus recti................ 284
Prospectus of the Crown Company 557
Ptyalism, Mr. Arnott on........ 153

Pubes, separation of, cases of.... 526
Puerperal fever, observations on 243
Puerperal Peritonitis, Mr. Davies on .......................... 503
Puerperal irritability, Mr. Walier on .............................. 503
Puerperal fever of Vienna, report of 509
Puerperal neuritis .............. 523
Puerperal neuritis its varieties .. 524
Pulse, observations on the..... 422
Purpura hæmorrhagica ......... 193
Purulent ophthalmia of infants.. 220
Pyramus frigate, fever in........ 11
Quarantine, Dr. Maclean on .... 18
Quinine, sulphate of, in typhus.. 237
Quinine, sulphate of, its powers.. 488
Recto vesical lithotomy ........ 473
Reid, Dr. on epilepsy ........... 205
Remuneration, medical, remarks on ............................ 246
Report of the small-pox Hospital, by Dr. Gregory................ 551
Researches, in physiology, by Dr. Blundell .................... 400
Resuscitation, Dr. Roesler on .. 440
Rete mucosum, anatomy of .... 32
Retention of urine in pregnancy, case of ..................... 523
Retention of urine, cured by puncture .................... 477
Rheumatism treated by a boiling bath ......................... 302
Rheumatism, observations on its metastases, by Dr. Cox ...... 428
Rhone and Rhine, the waters of.. 443
Rupture of dropsical ovary, cases of ........................... 404
Ryall, Mr. Isaac, on purulent ophthalmia .................. 220
Sanders, Mr. H. on puerperal fever ........................ 244
Sand rock spring, salts of the .. 551
Scout sloop of war, fever in . .. 12
Scrofula, Dr. Alison on ......... 98
Secale cornutum, Dr. Hosack on 59
Sewel, Mr. on contagious glanders 249
Skin, Mr. Chevalier on ......... 29
Skin, Mr. Plumbe on ........... 29
Skin, M. Alibert on ............. 29
Snuff-taking, Dr. Kinglake on .. 250
Softening of the stomach ...... 173
Sour bread, a supposed cause of goitre......................... 442
Spasmodic stricture of the anus.. 300
Spasms in children, a peculiar kind of ...................... 452
Spinal marrow, construction of.. 151
Spine, Mr. Bampfield on ...... 112
Spleen, two cases of loss of...... 403
Staphyloma cured by tapping .. 475
Stoker, Dr. on fever .......... 1
Stomach, curious disease of .... 170
Stomach, tumours in .......... 174
Striking cures ................. 234
Subjects, on the preservation of.. 248
Sulphate of quinine, M. Bally on 530
Suppression of urine .......... 183
Sutleffe, Mr. his book of experience ....................... 25
Sympathetic affections of the chest ........................ 72
Symptomatology, Dr. Buchan on 413
Syphilitic ophthalmia, Mr. Hewson on ..................... 305
Tacheron, pathological researches 326
Tartar emetic ointment in epilepsy ........................ 486
Teeling, Dr. on suppression of urine ....................... 183
Tetanus, Mr. Carmichael on.... 193
Texture, cellular, inflammation of ........................... 335
Thoracis paracentesis .......... 479
Tinct. ferri muriatis .......... 359
Tinctura colchici, Mr. Bushell on 531
Tobacco, in dysentery, Dr. O'Beirne on ................ 495
Tongue, its different appearances 424
Tonsils, enlargement of—Dr. Belcher ...................... 217
Tracheotomy in cynanche trachealis ........................ 215
Tracheotomy, Mr. Carmichael .. 223
Transfusion, Dr. Blundell's ideas on ........................... 410
Treatment of Herniæ .......... 211
Treatment of iritis ............ 469
Trephine, Mr. Wise on the use of 536
Tumour, of great size, removed.. 219
Tumours, hæmorrhoidal......... 376
Typhus, sulphate of quinine in.. 237
Ulcers of the cornea, Mr. Melin on .......................... 472
Ulcers, origin of tight bandaging for ......................... 532
Urine, suppression of .......... 183
Uterus, irritable, case of........ 512
Uwins's, Dr. compendium of medicine........................ 382
Vein—air introduced to the heart by ........................ 218
Veins, inflammation of ........ 161
Veins, a case of inflammation of 447
Venesection, dangers of ........ 336
Venesection, in rheumatism.... 429
Venesection, in repeated abortion 519
Vilermay, M. on inflammation of appendix cœci ............... 200
Vital power, M. Martinet on.... 433
Wade, Mr. on fever............ 1
Wedeymeyer, Dr. on paracentesis thoracis.................... 213
Westminster Medical Society, its report ..................... 558
Willis, Dr. on insanity.......... 360
Wise, Mr. on the trephine...... 536
Wood, Mr. on purpura hæmorrhagica ................... 196
Yellow fever, its distinctions ... 12
Zink, Dr. on iodine............. 229

